# Liquid-Crystal-Elastomer-Actuated Reconfigurable Microscale Kirigami Metastructures

**DOI:** 10.1002/adma.202008605

**Published:** 2021-05-13

**Authors:** Mingchao Zhang, Hamed Shahsavan, Yubing Guo, Abdon Pena-Francesch, Yingying Zhang, Metin Sitti

**Affiliations:** Key Laboratory of Organic Optoelectronics and Molecular Engineering, of the Ministry of Education, Department of Chemistry, Tsinghua University, Beijing 100084, China; Physical Intelligence Department, Max Planck Institute for Intelligent Systems, 70569 Stuttgart, Germany; Physical Intelligence Department, Max Planck Institute for Intelligent Systems, 70569 Stuttgart, Germany; Department of Chemical Engineering and Waterloo Institute for Nanotechnology, University of Waterloo, Waterloo, ON N2L 3G1, Canada; Physical Intelligence Department, Max Planck Institute for Intelligent Systems, 70569 Stuttgart, Germany; Physical Intelligence Department, Max Planck Institute for Intelligent Systems, 70569 Stuttgart, Germany; Department of Materials Science and Engineering, Macromolecular Science and Engineering, Robotics Institute, University of Michigan, Ann Arbor, MI 48109, USA; Key Laboratory of Organic Optoelectronics and Molecular Engineering, of the Ministry of Education, Department of Chemistry, Tsinghua University, Beijing 100084, China; Physical Intelligence Department, Max Planck Institute for Intelligent Systems, 70569 Stuttgart, Germany; Institute for Biomedical Engineering, ETH Zürich, Zürich 8092, Switzerland; School of Medicine and School of Engineering, Koç University, Istanbul 34450, Turkey

**Keywords:** kirigami, liquid crystal elastomers, reconfigurable metastructures, two-photon polymerization, wireless microscale devices

## Abstract

Programmable actuation of metastructures with predesigned geometrical configurations has recently drawn significant attention in many applications, such as smart structures, medical devices, soft robotics, prosthetics, and wearable devices. Despite remarkable progress in this field, achieving wireless miniaturized reconfigurable metastructures remains a challenge due to the difficult nature of the fabrication and actuation processes at the micrometer scale. Herein, micro-scale thermo-responsive reconfigurable metasurfaces using stimuli-responsive liquid crystal elastomers (LCEs) is fabricated as an artificial muscle for reconfiguring the 2D microscale kirigami structures. Such structures are fabricated via two-photon polymerization with sub-micrometer precision. Through rationally designed experiments guided by simulations, the optimal formulation of the LCE artificial muscle is explored and the relationship between shape transformation behaviors and geometrical parameters of the kirigami structures is build. As a proof of concept demonstration, the constructs for temperature-dependent switching and information encryption is applied. Such reconfigurable kirigami metastructures have significant potential for boosting the fundamental small-scale metastructure research and the design and fabrication ofwireless functional devices, wearables, and soft robots at the microscale as well.

## 1 Introduction

Metamaterials or architected materials consist of engineered periodic arrangements of building units^[1,2]^ and possess exotic properties and functionalities that do not exist in nature, such as negative refractive index,^[3]^ negative Poisson’s ratio,^[4]^ and mechanical cloaking.^[5]^ Reconfigurable metastructures, which can adaptively alter their complex shapes or morphologies,^[6]^ form an active subcategory of the architected materials with significant potential in developing deployable and dynamically tunable structures for advanced technologies, such as optical lenses,^[7]^ soft robots,^[8]^ smart wearables,^[9]^ and biomedical devices.^[10]^ Kirigami structures are ideal platforms for constructing reconfigurable metastructures based on prescribed periodic cuts on thin sheets or films. A great variety of kirigami structures can be achieved by creating different shapes for cuts, such as linear, triangular, or square cuts. As such, kirigami, in recent years, has been an attractive source of inspiration for the development of morphing metastructures.^[11–14]^

Most of the current research on morphable kirigami metastructures focuses on macroscopic structures.^[14–16]^ Indeed, the established design rules and algorithms for the fabrication and programmed shape morphing of kirigami metastructures are still limited to macroscopic scales.^[17]^ Fabrication, programming, and reversible actuation of metastructures at nano- and microscales have been hitherto a grand challenge impeding the progress of reconfigurable metastructures in such small scales. There have been a few remarkable reports, which mainly relied on complex and costly fabrication techniques, such as focused ion beam irradiation,^[18,19]^ soft lithography,^[20]^ and multi-step microfabrication,^[21,22]^ to construct small-scale metastructures. Recent advances in microscale 3D printing based on two-photon polymerization (2PP)^[23–27]^ have facilitated the fabrication of small-scale metastructures with high freedom yet exceptional resolution down to 100 nm.^[28,29]^ Nevertheless, the actuation mechanisms in most of the previous reports are still limited to a few methods, such as microscale physical probes^[30]^ and solvent/ion-induced swelling/shrinking,^[1,31]^ hindering their reversibility, controllability, and programmability.

To build the small-scale reconfigurable metastructures, a suitable wireless remote actuation strategy is key in bridging the gap between macroscopic stimuli and small-scale metastructures. Artificial muscles from liquid crystal elastomers (LCEs) can offer promising solutions for the remote actuation of architected microstructures. For example, Woska and co-workers have recently employed LCEs to realize a platform for tunable microscale photonic architectures using 3D laser printing.^[32]^ LCEs respond to external stimuli, such as heat and light, typically through a variety of large and programmable shape morphing behaviors.^[33–38]^ Shape morphing of LCEs is mainly dictated by their molecular alignment, the so-called director field.^[23,36]^ Upon exposure to an external stimulus, such as heat, LCEs usually contract along their local director field while expanding in an orthogonal direction.^[33,36,39,40]^

These properties make LCEs ideal artificial muscles with programmable and reversible actuation capabilities at the microscale, which can power the transformation of kirigami microstructures. The director field of LCEs can be manipulated with microscale resolution through various techniques, such as micro-rubbing,^[41]^ plasmonic photopatterning,^[42]^ and magnetic alignment.^[43]^ Such precise molecular alignment methods allow for programmable and precise shape morphing even at submillimeter scales.^[41,44–46]^

In this work, we have leveraged the programmable and responsive properties of LCEs, and exceptional fabrication capabilities of the 2PP technique to achieve microscale programmable and reconfigurable metasurfaces. We used LCEs as artificial muscles to remotely actuate kirigami microstructures fabricated by the 2PP. We explored their different thermoresponsive transformation behaviors corresponding to rationally designed geometrical parameters through experiments and simulations. We demonstrated conceptual applications, such as temperature-dependent switching and information encryption based on our LCE-actuated kirigami metastructures. However, we believe our strategy for the design, fabrication, and actuation of reconfigurable microscale metastructures has great potential in boosting the development of fundamental studies and emerging technologies such as wireless functional devices, wearables, and soft robots at the microscale.

## 2 Results and Discussion

**Figure 1:**
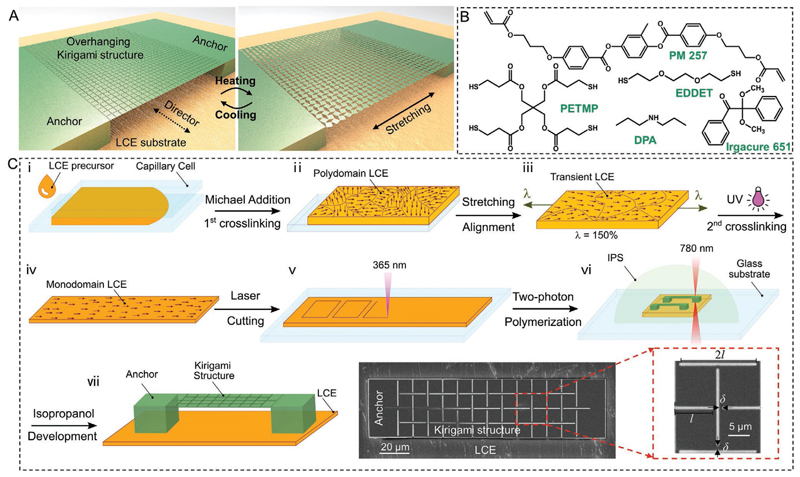
Concept and fabrication of LCE-actuated kirigami microstructures. A) Schematic illustration showing the concept of overhanging 3D-printed kirigami microstructures stretched by a heated LCE film (with a director field perpendicular to the stretching direction) attached firmly to the microstructure bases through two micropost anchors. B) Chemical structure of the used LCE film precursor. C) Microfabrication process of uniaxially aligned LCE substrates followed by two-photon polymerization-based 3D printing of the overhanging kirigami microstructures. The insets show scanning electron microscopy (SEM) top-view images of a kirigami structure on the LCE substrate.

Our design of microscale programmable and reconfigurable metasurfaces is schematically shown in Figure 1A, where an overhanging kirigami microstructure is 3D printed via the 2PP technique and anchored by two microposts to a uniaxially aligned LCE film with a large surface area. The LCE backing substrate acts as an artificial muscle that can be reversibly actuated in a programmable manner by an external stimulus, such as heat. In this design, the kirigami microstructure can be remotely stretched or contracted if the elastic energy is stored or released during the thermal deformation (e.g., uniaxial contraction, in the uniaxially aligned LCE artificial muscle case) of the backing LCE is properly harvested and transferred to it. In the majority of current LCEs, the molecular disorder induced by heat results in contraction parallel and expansion perpendicular to the local director field. As such, we can precisely program the mode of deformation of the kirigami microstructure before the fabrication and by adjusting the orientation of the strain fields of the microstructure relative to the director of the LCE substrate. In addition, our strategy relies on the balance between the output work generated (*W*_out_) by the LCE substrate and the energy required to stretch the kirigami microstructure (*W*_in_), as well as, the efficiency of the energy transfer (*η*) by the two anchoring microposts. As a key metric for active materials, the output work *W*_out_ is determined by the actuation strain *ε* and material elastic modulus *E* (W_out_∝ *vε*^2^*E*, where *v* is the original volume), which are typically mutually exclusive.^[47,48]^ As such, it is crucial to carefully choose the LCE formulation and optimize the geometrical parameters of both kirigami microstructures and microposts to realize the remotely driven and reversible transformation of the microstructures.

In our preliminary investigation to choose the proper active liquid-crystal-based substrate as an artificial muscle, we examined the two extensively studied systems of glassy acrylate-based liquid crystal networks (LCNs)^[36,49]^ and thiol-acrylate-based LCEs.^[37,38]^ We were able to create thin films with various predetermined director fields and mechanical properties from both material systems. However, initial results confirmed that glassy LCNs could not produce sufficient output work for the transformation of overhanging kirigami microstructures. We believe that the inherently smaller thermal strain of uniaxially aligned glassy LCNs and softening at elevated temperatures adversely affect the output work of glassy LCNs. Therefore, as shown in Figure 1B, we used thiol-acrylate LCE systems, which typically have large strains and Young’s modulus, throughout this study.

The schematics of the synthesis and molecular alignment of the LCE film followed by the 3D printing of kirigami micro-structures are shown in Figure 1C-i-vii. i) In brief, the LCE pre-cursor mixture was injected into capillary cells with a ≈200 μm gap and left at room temperature for at least 5 h to render the Michael addition (the first step of cross-linking). ii–iv) Then, the cell was cleaved, and the resulting swollen gel was dried in an oven under the vacuum at 50 °C for at least 6 h. The dried polydomain LCE film was then uniaxially stretched to ≈150% of its original length for planar molecular alignment along the stretching axis. The second step of cross-linking was carried out by exposing the stretched LCE film to ultraviolet light (UV). v) The fully cross-linked monodomain LCE film with a thickness of ≈100 μm was then laser-cut into 1.5 mm × 1.5 mm square films that would act as the artificial muscle. Finally, vi) square-cut kirigami microstructures with different geometries and sizes were 3D printed on top of LCE films via the 2PP of a commercially available photoresist (IPS780), as shown by the snapshots taken during the 3D printing of kirigami microstructures on the LCE film (Figure S1, Supporting Information). In this design, the areas polymerized by the laser will form rotating sheets and hinges, while the unpolymerized regions act as prescribed cuts of kirigami structures after development. Note that the bottom of anchoring two microposts must be printed deeply below the IPS780/LCE interface to create a strong adhesion between the LCE film and two posts, which ensures a high *η* and prevents undesirable delamination upon heating. Figure 1C-vii shows the schematic view and scanning electron microscope (SEM) images of a typical kirigami microstructure.

**Figure 2:**
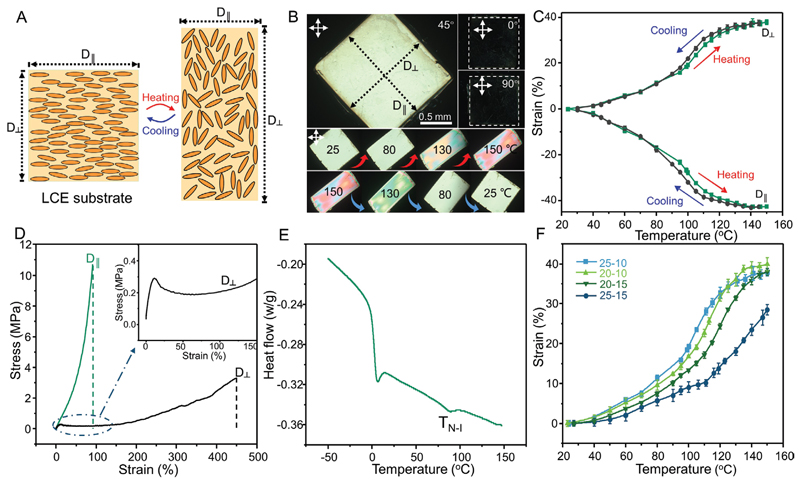
Characterization of the uniaxially aligned LCE film as the artificial muscle actuator. A) Schematics showing the mechanism of reversible deformation (contraction along the LCE director field and stretching perpendicular to the LCE director field, after heating) of the uniaxially aligned LCE film during heating and cooling cycles. B) Polarized optical microscopy (POM) images showing the uniaxially aligned LCE substrate under different observing angles and its reversible deformation during heating and cooling cycles. C) Thermal contraction and expansion measurements in the directions parallel (D_||_) and perpendicular (D_⊥_) to the director field of the LCE. D) Mechanical properties of the LCE substrate in the directions parallel and perpendicular to the LCE director field. E) Differential scanning calorimetry (DSC) characterization of the LCE film material. F) Thermal expansion (D_⊥_) of the LCE materials with different precursor formulations.

Molecular disorder imposed by heating the uniaxially aligned LCE film typically leads to large thermal contraction and expansion in the directions parallel (D_||_) and perpendicular (D_⊥_ to their director fields, respectively (Figure 2A). Hence, the pre-determined stretching axis of the kirigami structures was set to be perpendicular to the LCE director field in all our experiments. The native and thermally deformed shapes of a piece of uniaxially aligned LCE film synthesized in this work under the crossed-polarized microscope are shown in Figure 2B and Movie S1 (Supporting Information). The sample experienced a large and reversible anisotropic thermal deformation when heated up. Hysteresis was negligible as the sample recovered to its initial shape and size after cooling (Figure 2B,C). To optimize the thermomechanical properties of LCEs for our application, we examined previously reported formulations^[38,39]^ with different amounts of diacrylate and tetra-thiol cross-linkers, toward increasing the strain without sacrificing the modulus. We chose LCE-25-10 with 25% tetra-thiol (thiol functional groups from pentaerythritol tetra(3-mercaptopropionate) (PETMP)) and 10% excess acrylate (relative to all the thiol functionalities) (Table S1, Supporting Information). Mechanical properties of LCE-25-10 were measured at room temperature, which indicated large mechanical anisotropy parallel and perpendicular to the LCE director field (Figure 2D). Phase change behavior of LCEs, as a determining factor in their thermomechanical properties, was characterized by differential scanning calorimetry (DSC). Results in Figure 2E show a pronounced phase change from nematic to isotropic phase for the LCE-25-10 at *T*_NI_ ≈80 °C. Some samples with other formulations did not show much difference in their phase evolution behavior with temperature and some had even less distinct peaks for their *T*_NI_. Thermal deformation data for all tested samples are presented in Figure 2F, which confirms that LCE-25-10 had the highest thermal strain up to 120 °C.

**Figure 3:**
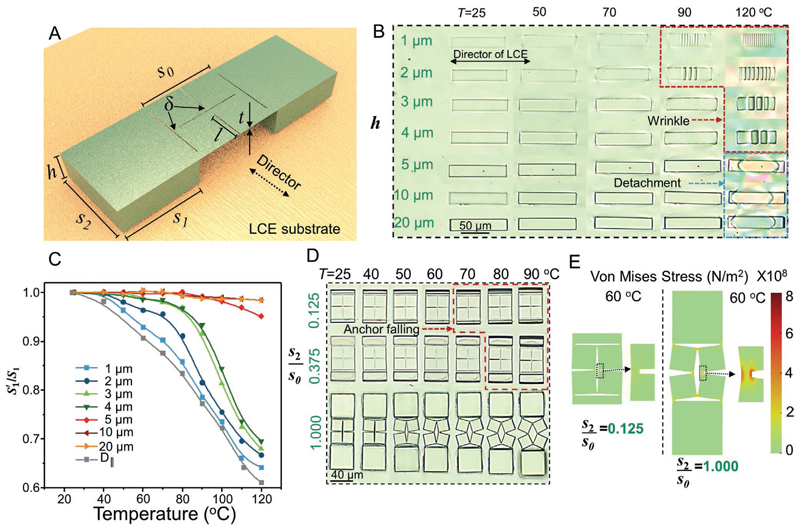
Controlling the shape transformation of the kirigami microstructures as a function of the geometrical design of two anchoring posts. A) Schematic illustration of a single kirigami microstructure unit printed on the LCE substrate. B) POM images showing the effect of anchoring post height upon heating. C) Width change, *S*′_1_/*S*_1_ of two anchoring posts with different heights when heated. D) Snapshots showing the transformation of three kirigami units with different lengths (*S*_1_) of anchoring posts at different temperatures. E) Simulation results showing the stress distribution of two anchoring posts with different lengths at 60 °C.

We then explored the relationship between shape-morphing behaviors of kirigami microstructures and various geometrical parameters. Geometrical parameters of anchoring posts and overhanging kirigami microstructures (including the size and shape of the cuts and hinges) all contribute to the shape-transformation behaviors of the 3D-printed kirigami microstructures. We systematically changed these parameters, shown in Figure 3A, for isolated anchoring posts and basic kirigami units. Figure 3B shows the impact of the height of the anchoring posts (*h*) on the deformation behavior of the IPS780/ LCE bilayers. Due to the large thermomechanical mismatch between IPS780 and LCE, raising the temperature of the systems with thinner IPS780 layers led to the appearance of surface wrinkling as a result of Euler structural instabilities^[50]^ (highlighted with red dash line in Figure 3B), particularly for samples with *h* < 5 μm. Such local out-of-plane deformation of IPS780 layers also led to a significant decrease in the width (*S*_1_) of the anchoring posts due to the thermal shrinkage of the LCE film along its director field (Figure 3C). The shrinkage of the anchoring posts along their width frustrates the desired stretching of the overhanging kirigami structures, thus hindering the transformation. This was not the case for anchoring posts with larger heights (*h* > 5 μm) that maintained their structural integrity and original width. In turn, at very high temperatures, we observed delamination from the interface and reduction of the IPS780/LCE interfacial area (highlighted with blue dash line in Figure 3B). The threshold temperature for such instability was well above the *T*_NI_ of our LCE. As such, to eliminate the adverse effect of the posts’ shrinkage along their width and to minimize the reduction of the interfacial area, the height of anchoring posts was kept higher than 5 μm throughout this work. The mode of buckling and characteristics of wrinkles, such as their wavelength or amplitude, can be described in the framework of the Euler-Bernoulli bilayer beam theory.^[51]^

The length of anchoring posts (*S_2_*) is another key parameter that determines the overall adhesion, effective contact area (*S*_1_ · *S*_2_), and consequently the efficiency of energy transfer between the kirigami structure and the LCE film. Kirigami structure could not be stretched and transformed even at high temperatures when the ratio between the length of their anchoring posts and the main square unit (*S*_2_/*S*_0_) was smaller than 0.375. At high temperatures, these samples detached from the LCE film, which can be seen by shadows around the falling anchoring posts highlighted with a red dashed line in Figure 3D. Samples with longer anchoring posts, such as those with *S*_2_/*S*_0_ = 1, maintained their adhesion at high temperatures, resulting in an efficient energy transfer from the LCE film to the kirigami structure and high shape transformation. To better understand the effect of *S*_2_ on the overall stress created on the kirigami structure, we conducted finite element simulations of a basic unit. As shown in Figure 3E, anchoring posts with small *S*_2_ could not provide sufficient stress to stretch the kirigami structure. On the contrary, samples with larger *S*_2_ generated sufficiently large stress at hinges, resulting in the rotation of elementary squares to wider angles.

**Figure 4:**
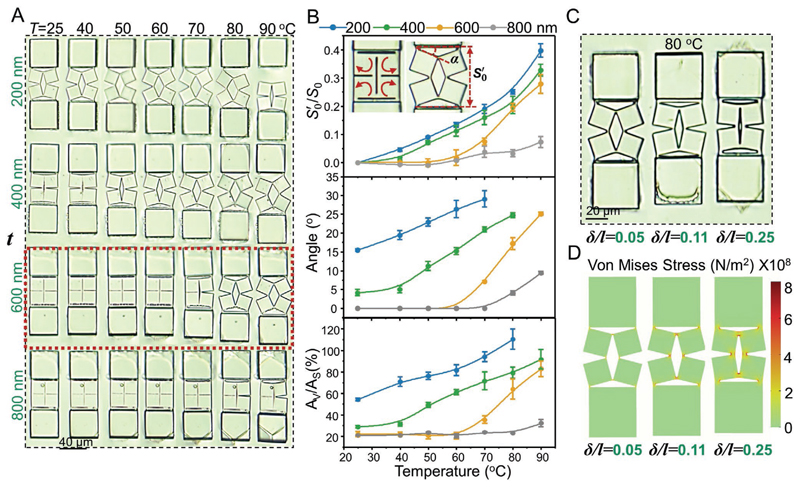
Effect of the geometrical parameters of the kirigami microstructures on their shape transformation. A) Snapshots showing the transformation of four kirigami units with different thicknesses (t) of the overhanging kirigami structure at different temperatures. B) Quantitative analysis of the effect of *t* of the overhanging kirigami structures on the evolution of the length (*S*_0_), turning angle (α), and generated void area (*A*_v_) at different temperatures. C) Images of three kirigami units with different length ratios of joint width to square edge length (*δ/l*) at 80 °C. D) Simulation results showing the stress distribution of three basic units with different ratios of *δ/l* at 80 °C.

In addition to the geometrical parameters of anchoring posts, the thickness (*t*) of overhanging kirigami structures have a significant impact on the extent and configuration of the shape transformations. Since the required work to actuate the kirigami structures is proportional to their prescribed volumes (*W*_in_ ∝ *vε^2^E*), the thickness should be rationally designed to meet *W*_out_ · **η* > W*_in_ condition for the shape transformation, but *W*_out_ · *η* ≫ *W*_in_ should be avoided in case of causing rupture to the kirigami structures. As shown in Figure 4A, we were able to stretch structures with *t* ≤ 600 nm by raising the temperature above the *T*_NI_ of the LCE film. The generated stress in the samples with *t* ≤ 400 nm, caused the rupture of hinges, as it surpassed the fracture limit of the IPS780 material. Note that the thin kirigami structures (*t* < 600 nm) show a premature shape-transformation even before heating. Such a premature shape transformation can be attributed to the external stress caused by the slight swelling of LCE films during the development process in isopropyl alcohol (Figure S2, Supporting Information), causing unintended stretching of kirigami structures. On the contrary, structures with *t* ≥ 800 not only maintained the intended closed-form initial configuration, but also resisted the stress generated by the expansion of the LCE film when heated. As such, we set the thickness of kirigami structures to 600 nm as an optimal value for the shape transformation of kirigami microstructures over a wide range of temperature changes (highlighted with the red dash line in Figure 4A). Samples with this optimal thickness also resisted the swelling of the LCE film to prevent the pre-stretching, and experienced shape transformation only after they reached their threshold stress in the vicinity of *T*_NI_.

We quantitatively analyzed the effects of the kirigami structure thickness on the ratio between their real-time length (*S*′_0_) and the original length (*S_0_*), the rotation angle of their square subunits (α), and the ratio between the void area (*A*_v_) and the original solid area (*A*_s_), as shown in Figure 4B. Variation of these parameters with temperature shows that structures with *t* < 600 nm undergo a gradual and relatively monotonic incremental behavior. This trend did not hold for structures with the optimal thickness (600 nm), where a relatively sharper increase in all parameters began to appear after ≈60 °C. This transient behavior at a threshold temperature enables on-demand temperature-sensitive switching of shape transformation. Samples with *t* > 600 nm showed a very small increase in the three values even at higher temperatures.

The last design parameter we studied was the ratio between the length of kirigami metastructure cuts (*l*) and hinges (*δ*), owing to the great impact they have on the extent of shape transformation.^[52]^ For this, we varied *δ/l* and monitored the evolution of their shape transformation with temperature changes (Figure 4C and Figure S3: Supporting Information). Kirigami structures with smaller *δ/l* (for example, ≈0.05) easily transformed when heated, while those with a large *δ/l* (for example, ≈0.25) resisted the stretching induced by the thermal deformation of the LCE film and experienced transformation at temperatures (≈90 °C) higher than *T*_NI_. Samples with *δ/l* ≈ 0.11 showed reversible shape transformation at temperatures closer to *T*_NI_ at ≈70 °C. Figure 4C shows snapshots of the samples with these three ratios at 80 °C alongside their corresponding simulation results in Figure 4D. Since the long hinges resist the stretching induced by the deformation of LCE substrates, their local stress is larger than that of short hinges.

**Figure 5:**
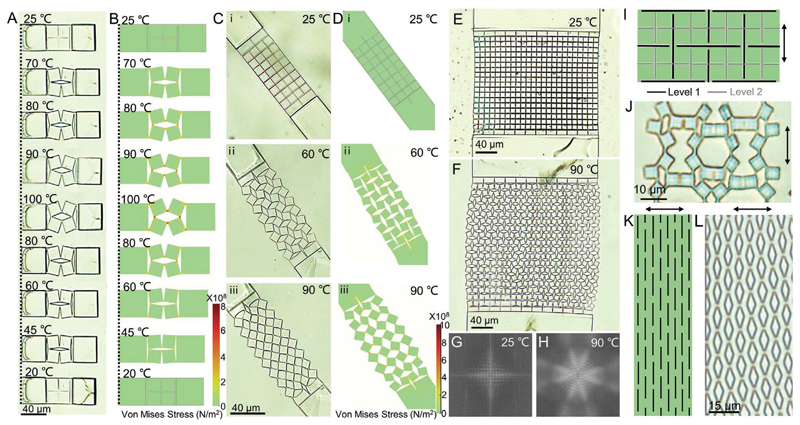
Shape morphing of the kirigami microstructures with different unit numbers and motifs. A) Evolution of a single kirigami unit with optimized geometrical parameters showing reversible shape-transformation during heating and cooling cycles. B) Simulation results showing the stress distribution of the single kirigami unit during heating and cooling cycles. C) Long-strip kirigami structure with 2×4 units (32 hinged squares) and its configuration snapshots at different temperatures. D) Simulation results of the long-strip kirigami structure at different temperatures. E,F) A kirigami structure design with 9×12 units (432 hinged squares) at 25 °C (E) and 90 °C (F). G,H) Fast Fourier transformation diffraction patterns of the closed (G) and open (H) configurations at 25 and 90 °C, respectively. I) Schematic illustration showing a kirigami structure with the embedded -level-2 cuts. J) Configuration of the kirigami structure with embedded level-2 cuts at 90 °C. K) Schematic illustration showing another kirigami structure design with linear cuts. L) Configuration of the kirigami structure with linear cuts at 90 °C after LCE film-based stretching.

Using the optimal design parameters, we could elicit reversible shape transformation of single-unit kirigami microstructures by consecutive heating and cooling cycles (Figure 5A and Movie S2: Supporting Information). Moreover, Figure 5B shows an agreement between our simulation and experimental results. Next, we investigated the applicability of the obtained optimal parameters in the design, fabrication, and transformation of kirigami structures with a variety of sizes and motifs. Figure 5C shows the thermally induced shape transformation of a kirigami microstructure with 2x4 units (32 squares) at different temperatures, which closely matches the simulation results (Figure 5D). We could also fabricate and actuate kirigami structures with a much larger number of units (9x12 unit arrays), as shown in Figure 5E,F. Masses of repeating units generate large area meta-periodicity, which can be revealed by their power spectra (Figure 5G,H). Upon heating, the symmetry of the pattern changed along with the actuation of the LCE film. In addition to creating larger structures, our strategy can be applied to design and fabricate kirigami structures with other motifs. For example, the incorporation of higher-level cuts (level-2 cuts) into level-1 cuts (Figure 5I) generated a more intricate pattern (Figure 5J). Designing different cuts like linear cuts (Figure 5K) generated a mesh-like pattern upon actuation (Figure 5L). However, it should be noted that the maximum output strains by the LCE film limit the designing complexity and versatility of the kirigami microstructures. Our current LCE system fails to fully actuate kirigami structures with higherlevel cuts, such as level-3 cuts which require a maximum actuation of 79% strain.^[53]^ The development of LCE artificial muscles with higher output strains that enable the complete actuation of such complex configurations is a future work.

**Figure 6:**
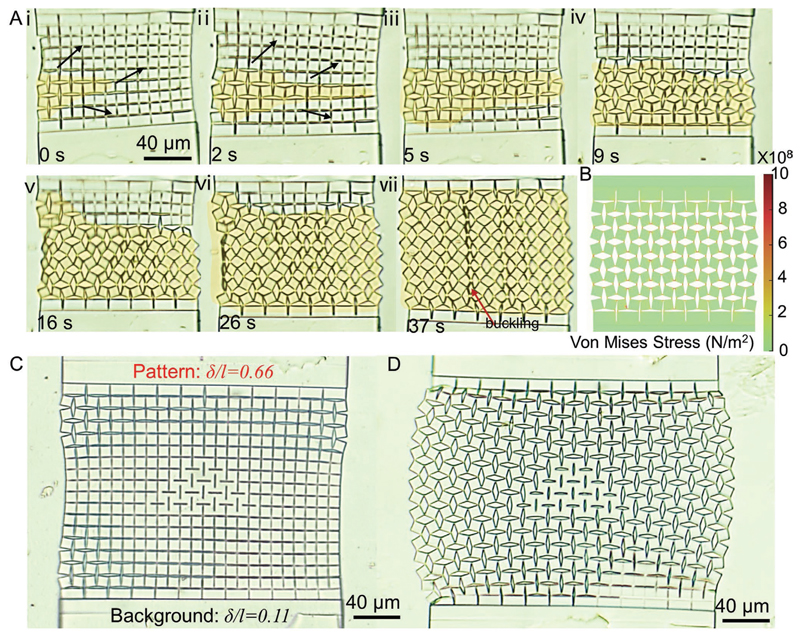
Proof-of-concept switching and data encryption application demonstrations of the proposed LCE-actuated microscale kirigami metastructures. A) A kirigami microstructure composed of 4×7 units for temperature-dependent shape-shifting from a “close” to an “open” configuration. The (i–vii) sequence shows the optical microscope image snapshots of the switching kirigami structures at a given time point, which resembles a wave propagation. The red arrow shows areas that experience out-of-plane buckling. B) Simulation result of the fully “open” kirigami microstructure composed of 4×7 units at 90 °C. C) An original configuration of the kirigami microstructure including 9×12 units with an embedded triangle pattern at 25 °C. D) A stretched configuration of the kirigami microstructure including 9×12 units with an embedded pattern of a triangle at 90 °C.

The controllable and scalable shape morphing behaviors of the kirigami microstructures show great promise for many potential applications. Figure 6A shows the shape transformation of a kirigami structure with 4×7 units upon relatively rapid exposure to a high temperature (90 °C). Square subunits open with a propagation speed of ≈4 μm s^–1^ in a fashion similar to the wave propagation of pop-ups in kirigami shells^[15]^ (Movie S3, Supporting Information) toward a fully opened configuration (Figure 6A-vii, and its simulation results in Figure 6B). In our construct, the propagation speed heavily depended on the heating source and heat transfer parameters. For our electrical heating systems, the heating speed was limited by the low thermal conductivity of the used backing glass substrate, and we believe that the wave propagation speed can be enhanced by using other contactless and fast heating methods, such as infrared light. Fast and controllable pattern transformation in kirigami microstructures has promise in phononic/photonic devices. For instance, the controllable reconfiguration of periodic microstructures has been previously used for phononic switching.^[23]^

Information encryption was also achieved by incorporating specific patterns into the complex kirigami structures. These patterns were meant to be visible at a certain temperature. Figure 6C,D shows the original and transformed configuration of a kirigami structure with an arbitrary shape (triangle) embedded in them, respectively. These specific patterns can be created by modifying *δ/l* in specific points. For example, we maintained *δ/l* at ≈ 0.11 as the background configuration and encrypted a triangle by locally adjusting *δ/l to* ≈0.66 (Figure 6C and Figure S5a: Supporting Information). After heating the structure to 90 °C, the squares in the region with *δ/l* ≈ 0.66 showed smaller rotation angles, revealing the encrypted triangular pattern on the background with larger rotation angles (Figure 6D). The visibility of encrypted patterns crucially depends on the contrast between them and the background, which decreases with *δ/l* values. Indeed, no discernible pattern was observed for samples with *δ/l* values comparable to that of backgrounds, even at elevated temperatures (Figure S5b-d, Supporting Information).

## 3 Conclusions

We have demonstrated proof-of-concept programmable and reconfigurable metasurfaces at the microscale by using LCE artificial muscle films to thermally actuate kirigami microstructures 3D-printed via the 2PP technique. We optimized the output actuation strain of the LCE film by varying the formulation of the precursor, i.e., the amount of diacrylate and tetra-thiol cross-linkers. We further analyzed the relationship between the geometrical parameters of the kirigami structures on the LCE film and their thermal shape transformation behaviors through experiments and simulations. We have demonstrated the scalability of these morphable kirigami metastructures, from one unit to periodic arrays with over 100 units. Finally, we explored the potential applications of devices based on such kirigami metastructures, such as thermal switching and information encryption. Our proposed strategy enables the design, modeling, fabrication, and behavior prediction of programmable reconfigurable micro-metastructures, by translating the existing knowledge of macroscopic kirigami metastructures to microscale. Furthermore, our strategy paves the way to a host of potential applications, such as tunable phononic/photonic crystals, optoelectronics, microelectromechanical systems, biomedical devices, camouflage, and soft microrobots.

## 4 Experimental Section

### Synthesis of the Uniaxially Aligned LCE Film

The LCE precursor is adopted and modified from previous literature and its formulation is shown in Figure 1B.^[38]^ 4-Bis-[4-(3-acryloyloxypropypropyloxy) benzoyloxy]-2-methylbenzene (RM257) was used as monomer; 2,6-di-*tert*-butyl-4-methylphenol (BHT) was used as thermal inhibitor; 2,2-dimethoxy-2-phenylacetophenone (Irgacure-651) was used as photoinitiator; 2 wt% dipropylamine (DPA) in chloroform solution was used as the catalyst for the Michael addition reaction; 2,2-(ethylenedioxy)diethanethiol (EDDET) was used as flexible chain extender; pentaerythritoltetra(3-mercaptopropionate) (PETMP) was used as crosslinker. RM 257 (500 mg), BHT (1.33 mg), and Irgacure-651 (3.77 mg) were dissolved into chloroform (0.20 mL) at 80 °C. Then EDDET (105.7 mg), PETMP (47.1 mg), and DPA solution (78.4 mg, 2 wt%) were quickly mixed to obtain the LCE precursor mixture.

The LCE precursor mixture was then quickly injected into a glass capillary cell with a ≈200 μm gap and left to dry at room temperature for at least 5 h to enable the Michael addition reaction. Then the swollen gel film was obtained after the cell was cleaved, and dried in a vacuum drying oven at 50 °C for at least 6 h. It should be noted the above-mentioned procedures should be implemented in a dark environment to prevent unwanted cross-linking. The dried polydomain LCE film was stretched to ≈150% of its original length to align the molecular orientation along its stretching axis. Then the molecular alignment was fixed by exposing the stretched LCE film to ultraviolet light for ≈15 min.

### Fabrication Procedures of the LCE-Film-Actuated Kirigami Microstructures

The obtained uniaxial LCE film was firmly fixed on an ultrasonically treated glass holder, and the LCE film was then laser-cut into arrays of squares with a side length of 1.5 mm by a laser cutter (ProtoLaser, LPKF Laser & Electronics AG). These LCE squares were left on the glass holder after peeling off the rest parts and then treated with an ultrasonic process each for 10 s in water and isopropyl alcohol, respectively. A drop of commercially available photoresist (IPS780, Nanoscribe GmbH) was put onto the LCE squares, and various kirigami structures with anchoring posts were then printed directly onto the LCE substrate via 2PP using a commercial Nanoscribe system (Nanoscribe GmbH). The adhesion was maximized between them during the fabrication due to the mechanical mismatch between the soft LCE substrates and stiff IPS780 anchoring posts. This proved to be crucial to enhance the energy transfer efficiency for transferring the stored elastic energy produced by the LCE actuation to the overhanging kirigami structures and achieving the desired shape transformation. This issue was addressed by writing the bottom of anchoring posts deep below the IPS780/LCE interface. Thus, the printed anchoring posts can crosslink with the surface unsaturated residues to form stable chemical bonds at the interface and firmly attach to the LCE film. As such, the printed structures are anchored well on the LCE substrate. It thus prevents being washed away during the development process or be detached during the later heating actuation.

After a 6 h development process in isopropyl alcohol, the uncured IPS780 precursor washed away and overhanging kirigami structures were then left on the LCE substrate. To release the LCE substrate from the glass holder, a 1 m NaOH aqueous solution was used to etch the glass substrate. After about 2 h, LCE-actuated kirigami structures were then released and floated on the solution surface for further usage.

### Characterization of Materials and the Actuation System

A polarized optical microscope (Zeiss; Axio Imager 2) was used to observe the bright field images and/or cross-polarized microscope images of LCE films and kirigami microstructures. A sealed heating chamber was assembled into the microscope to obtain their optical images at different temperatures. Differential scanning calorimetry (DSC2500; TA Instruments) was used to measure the phase transition temperatures of LCE materials with 10 °C min^–1^ heating and cooling rates. A rheometer (Discovery HB20; TA Instruments) was used to characterize the mechanical properties of LCE materials (stress-strain measurements were performed with a tension fixture at a rate of 200 μm s^–1^).

### Computational Modeling of the Reconfigurable Metastructures

Finite element analysis (COMSOL Multiphysics 5.4) was used to model the actuation of kirigami microstructures on the LCE film. Different geometric model of anchored kirigami structures on the LCE film was developed using 3Ds Max software (2016, Autodesk, Inc.). The mechanical properties of the LCE film were obtained from the strain-stress curves shown in Figure 2D and applied to the simulation. The overhanging kirigami structures were simulated with a Young’s Modulus of 4.6 GPa, a density of 1.2 g cm^–3^, and a Poisson’s ratio of 0.35.^[54]^

## Supplementary Material

Supporting Information is available from the Wiley Online Library or from the author.

Movie S1

Movie S2

Movie S3

Supplementary Material

## Data Availability

The data that support the findings of this study are available from the corresponding author upon reasonable request.
