# Supplementary material for: Liquid‐Crystal‐Elastomer‐Actuated Reconfigurable Microscale Kirigami Metastructures
Source: Adv Mater. 2021 May 13;33(25):2008605. doi: 10.1002/adma.202008605 (PMC7612660; doi:10.1002/adma.202008605)
Supplement: Supplementary file 1 — Supporting Information [file ADMA-33-2008605-s004.pdf]

© 2021 Wiley-VCH GmbH

ADVANCED MATERIALS

Supporting Information

for *Adv. Mater.,* DOI: 10.1002/adma.202008605

Liquid-Crystal-Elastomer-Actuated Reconfigurable Microscale Kirigami Metastructures

*Mingchao Zhang, Hamed Shahsavan, Yubing Guo, Abdon Pena-Francesch, Yingying Zhang, and Metin Sitti**

Supplementary Information

Liquid-Crystal-Elastomer-Actuated Reconfigurable Microscale Kirigami Metastructures

Mingchao Zhang[*]*, Hamed Shahsavan*[*]*, Yubing Guo*[*]*, Abdon Pena-Francesch, Yingying Zhang, and Metin Sitti*[+]

M. Zhang, Prof. Y. Zhang
Key Laboratory of Organic Optoelectronics and Molecular Engineering of the Ministry of Education, Department of Chemistry, Tsinghua University, 100084 Beijing, China

M. Zhang, Prof. H. Shahsavan, Dr. Y. Guo, Dr. A. P. Francesch, and Prof. M. Sitti
Physical Intelligence Department, Max Planck Institute for Intelligent Systems, 70569 Stuttgart, Germany

Prof. H. Shahsavan
Department of Chemical Engineering and Waterloo Institute for Nanotechnology, University of Waterloo, N2L 3G1 Waterloo, ON, Canada

Prof. M. Sitti
Department of Information Technology and Electrical Engineering, ETH Zürich, 8092 Zürich, Switzerland

[*] Equally contributing first authors

[+] Correspondence to: sitti@is.mpg.de

This PDF file includes:

- Supplementary Table 1
- Supplementary Figures 1 to 5
- Supplementary Movie 1-3 Captions

Other Supplementary Materials for this manuscript include the following:

- Movies S1 to S3

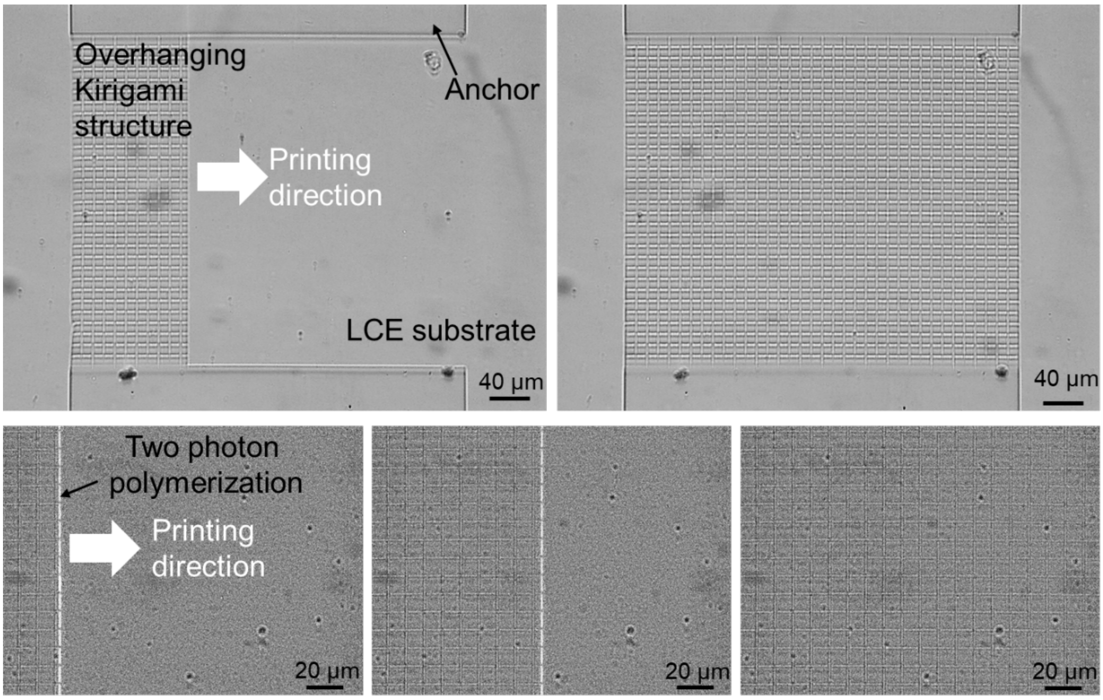

Supplementary Figure 1. Optical microscope images showing the 3D-printed kirigami microstructures on the LCE film/substrate.

Supplementary Table 1. Composition and thermal properties of LCE samples with different amount of thiol functional groups from PETMP and excess acrylate relative to thiol functionalities.

PETMP thiol content (%)	***Excess acrylate (%)***	***T_g (cooling, ^oC)***	***T_g (heating, ^oC)***	***T_{NI} (Heating, ^oC)***
25	10	-1.55	1.54	87.91
20	10	-2.71	0.34	93.97
20	15	-3.7	-0.17	92.94
25	15	0.10	2.74	102.06

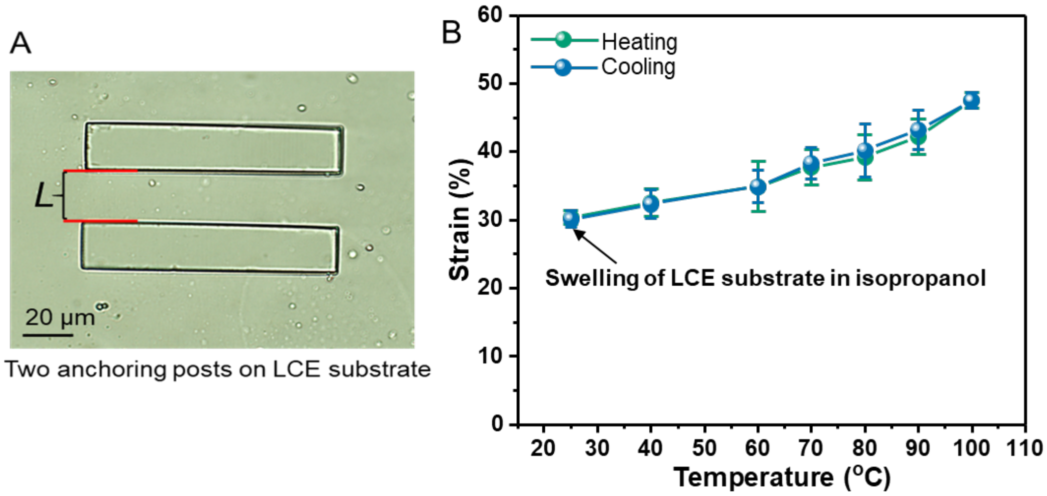

Supplementary Figure 2. Swelling characterization of the LCE film after development in the isopropanol solution. (A) Optical image showing two anchoring posts printed on the LCE film/substrate with a spacing of L. By measuring the distance of L before and after the development process in the isopropanol solution, a swelling strain of ~30% can be seen. (B) Thermal expansion (L/L_0) of the LCE film in a heating/cooling cycle, where L_0 is the original distance before development in isopropanol.

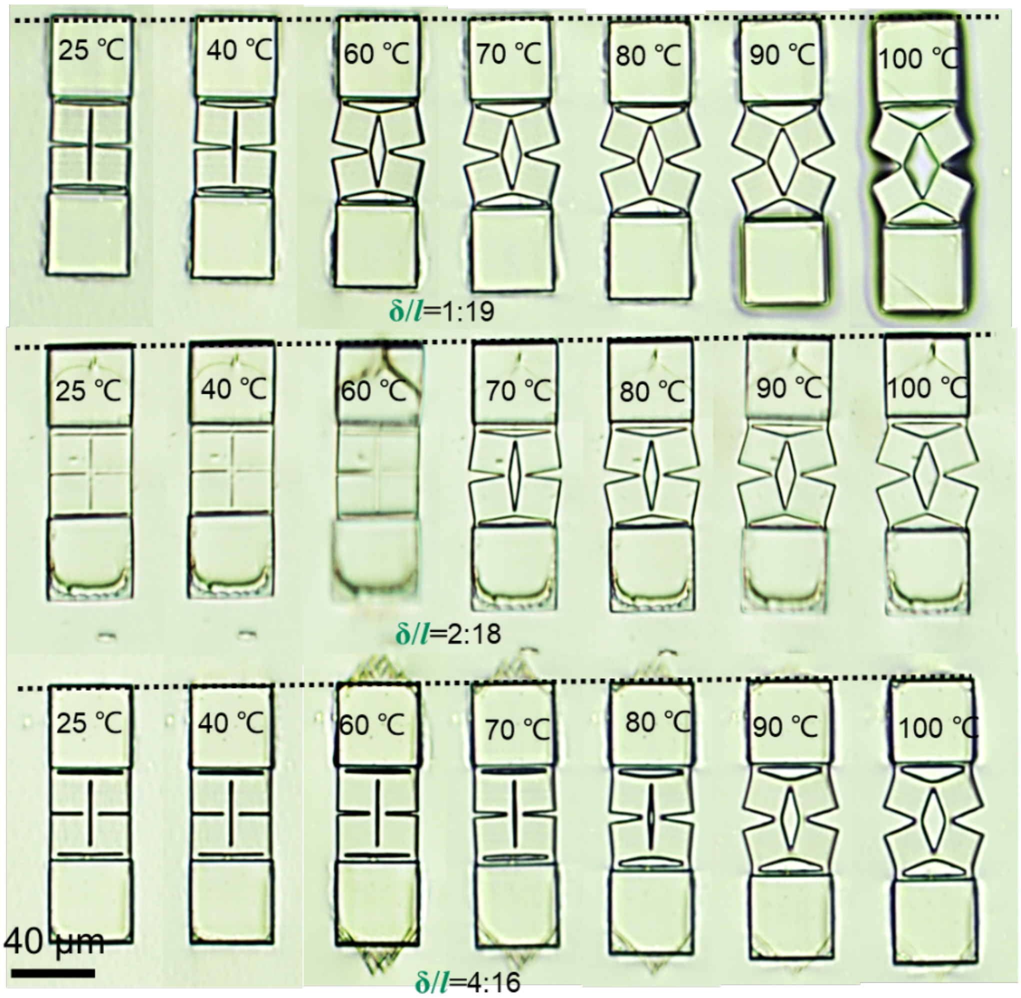

Supplementary Figure 3. The evolution of transformation of three kirigami units with different ratios of cut length to hinge length (*δ/l*) exposed to high temperatures.

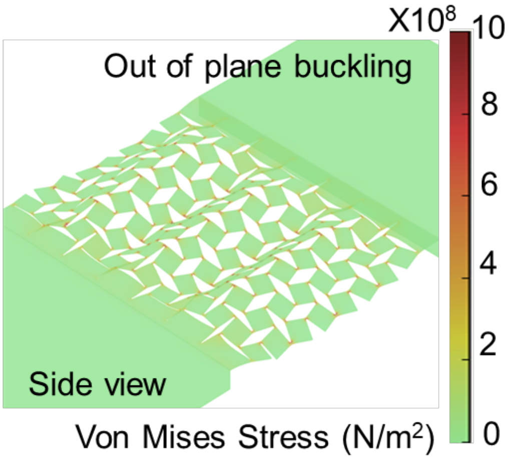

Supplementary Figure 4. Simulation result showing the side view of a fully opened kirigami microstructure composed of 4×7 units at 90 °C, showing an out-of-plane buckling. It is worth noting that unlike smaller kirigami structures (**Figure 5A**), larger kirigami structures are prone to experience irreversible thermal deformation due to the emergence of out-of-plane buckling (**Figure 6A** and **Supplementary Figure 4**)[53]. Such out-of-plane buckling increases the chances of kirigami structures touching and attaching to underlying LCE films. However, we think this problem can be alleviated by increasing the heights of the anchoring posts and introducing dislocations or defects to suppress the out-of-plane buckling[53]. The triangular pattern shown in **Figure 6B** frustrates such out-of-buckling, helping to enhance its reversibility.

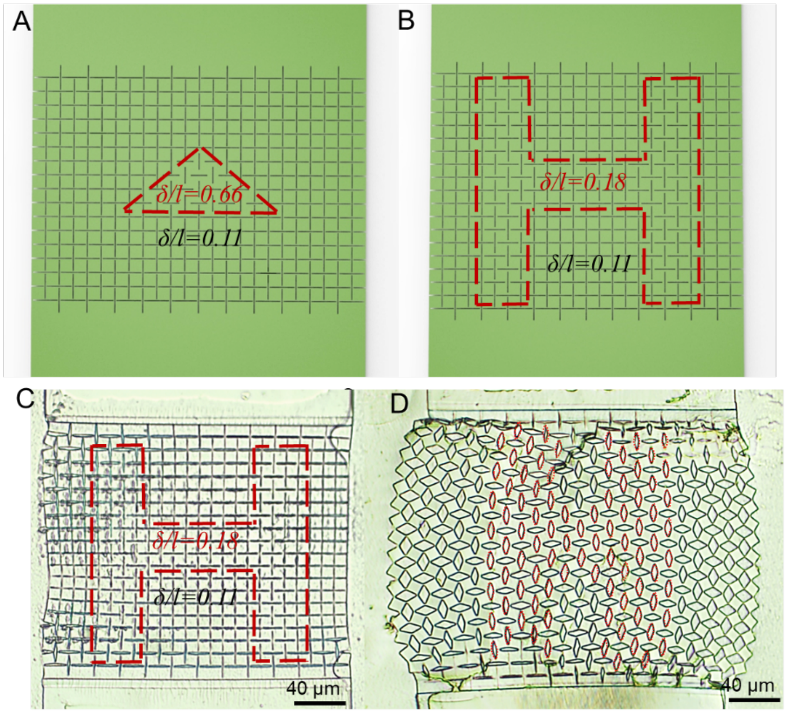

Supplementary Figure 5. Information encryption by embedding different values of *δ/l*. (A) A triangle with *δ/l=0.66* embedded in background configuration with *δ/l=0.11*. (B) A "*H*" letter with *δ/l=0.18* embedded in background configuration with *δ/l=0.11*. (C) An original configuration of the kirigami microstructure including 9×12 units with an embedded "H" pattern at 25 ^{o}C. (D) A stretched configuration of the kirigami microstructure including 9×12 units with an embedded pattern of a triangle at 90 ^{o}C.

Supplementary Movies

Supplementary Movie 1. Shape morphing of the liquid crystal elastomer film/substrate during heating and cooling.

Supplementary Movie 2. Evolution of the kirigami unit cell configuration during heating and cooling.

Supplementary Movie 3. Micromechanical wave propagation of the kirigami structure during heating (i.e., LCE film-based stretching).
